# Pharmacogenomics of Scopoletin in Tumor Cells

**DOI:** 10.3390/molecules21040496

**Published:** 2016-04-15

**Authors:** Ean-Jeong Seo, Mohamed Saeed, Betty Yuen Kwan Law, An Guo Wu, Onat Kadioglu, Henry Johannes Greten, Thomas Efferth

**Affiliations:** 1 Department of Pharmaceutical Biology, Institute of Pharmacy and Biochemistry, Johannes Gutenberg University, Mainz, Staudinger Weg 5, 55128 Mainz, Germany; seo@uni-mainz.de (E.-J.S.); saeedm@uni-mainz.de (M.S.); kadioglu@uni-mainz.de (O.K.); 2 State Key Laboratory of Quality Research in Chinese Medicine, Macau University of Science and Technology, Macau, China; klaw@must.edu.mo (B.Y.K.L.); wag1114@foxmail.com (A.G.W.); 3 Abel Salazar Biomedical Sciences Institute, University of Porto, Porto 4099-002, Portugal; heidelbergschool@aol.com; 4 Heidelberg School of Chinese Medicine, Heidelberg 69126, Germany

**Keywords:** ABC-transporter, cluster analysis, coumarin, herbal medicine, microarrays, multidrug resistance, phytotherapy

## Abstract

Drug resistance and the severe side effects of chemotherapy necessitate the development of novel anticancer drugs. Natural products are a valuable source for drug development. Scopoletin is a coumarin compound, which can be found in several *Artemisia* species and other plant genera. Microarray-based RNA expression profiling of the NCI cell line panel showed that cellular response of scopoletin did not correlate to the expression of ATP-binding cassette (ABC) transporters as classical drug resistance mechanisms (*ABCB1*, *ABCB5*, *ABCC1*, *ABCG2*). This was also true for the expression of the oncogene *EGFR* and the mutational status of the tumor suppressor gene, *TP53*. However, mutations in the *RAS* oncogenes and the slow proliferative activity in terms of cell doubling times significantly correlated with scopoletin resistance. COMPARE and hierarchical cluster analyses of transcriptome-wide mRNA expression resulted in a set of 40 genes, which all harbored binding motifs in their promoter sequences for the transcription factor, NF-κB, which is known to be associated with drug resistance. *RAS* mutations, slow proliferative activity, and NF-κB may hamper its effectiveness. By *in silico* molecular docking studies, we found that scopoletin bound to NF-κB and its regulator IκB. Scopoletin activated NF-κB in a SEAP-driven NF-κB reporter cell line, indicating that NF-κB might be a resistance factor for scopoletin. In conclusion, scopoletin might serve as lead compound for drug development because of its favorable activity against tumor cells with ABC-transporter expression, although NF-κB activation may be considered as resistance factor for this compound. Further investigations are warranted to explore the full therapeutic potential of this natural product.

## 1. Introduction

Despite the breathtaking progresses in cancer biology during the past decades, a cure from the disease is still not reality for the majority of patients. A main reason for this unsatisfactory situation is the development of drug resistance, which ultimately may lead to failure of chemotherapy and a fatal outcome for patients. Unfortunately, drug resistance occurs not only with long-established cytotoxic drugs, but also with the more recent small molecules and therapeutic antibodies, which are directed against specific targets in tumor cells [1]. Hence, the urgent struggle to identify and develop novel drugs continues. Drug resistance is frequently multifactorial and several mechanisms may account for unresponsiveness of tumors towards drugs. The multiplicity of mechanisms can be categorized as acting up- or down-stream of the actual drug target or at the target site itself [2,3].

The ATP-binding cassette (ABC) transporter P-glycoprotein (P-gp/*MDR1/ABCB1*) or the breast cancer resistance protein (*BCRP/ABCG2*) are well-known upstream acting mechanisms. They extrude multiple drugs out of cancer cells leading to multidrug resistance (MDR) phenomena [4,5]. Downstream mechanisms of drug resistance are for instance tumor suppressors and oncoproteins. After inactivation of target molecules (DNA, microtubules, DNA topoisomerases *etc.*), cytotoxic compounds influence DNA repair, cell cycle arrest, proliferation and apoptosis and thereby ultimately influence the fate of tumor cells.

Scopoletin is a coumarin compound, which can be found in many different medicinal plants, including *Scopolia* species (after which it was named) as well as species of the *Artemisia*, *Brunfelsia, Solanum*, *Mallotius* and other genus. Scopoletin is a constituent of *Artemisia annua* L. which is used for malaria treatment and also reveals activity towards cancer, schistosomasis and viral diseases [6,7,8,9,10,11]. We found high amounts of scopoletin in this plant, indicating that artemisinin may not be the only bioactive compound in *A. annua* [12]. Scopoletin is known for its cytotoxicity towards cancer cells [13,14,15]. It reveals antioxidant and anti-inflammatory features and induces apoptosis and autophagy [13,16,17].

Many xenobiotic and toxic natural products are detoxified from the body by ABC-transporters, e.g., at the blood brain barrier, gastrointestinal tract, liver, kidney and other organs [18]. In addition, P-gp and other ABC transporters are also important mechanisms of MDR in cancer [18]. Therefore, the question arises, whether or not scopoletin may be hampered in its cytotoxic action by ABC transporters.

In the present study, we investigated whether ABC transporters as classical MDR mechanisms also play a role in the response to scopoletin. Using the tumor cell line panel of the National Cancer Institute (NCI, USA), we addressed the question whether the cytotoxic activity of scopoletin may be compromised by the diverse mechanisms of MDR. In addition to ABC transporters (P-gp/*MDR1/ABCB1*, *ABCB5*, MRP1/*ABCC1*, BCRP/*ABCG2*), we also investigated oncogenes (*EGFR*, *RAS*) and the tumor suppressor gene, *TP53*. Furthermore, we performed *in silico* molecular docking studies of scopoletin to the drug resistance-mediating transcription factor NF-κB and its regulator IκB, as well as bioinformatic COMPARE and hierarchical cluster analyses of microarray-based transcriptomic mRNA expression data of the NCI cell lines (http://dtp.nci.nih.gov).

## 2. Results

### 2.1. Detection of Scopoletin in Artemisia annua

**Figure 1:**
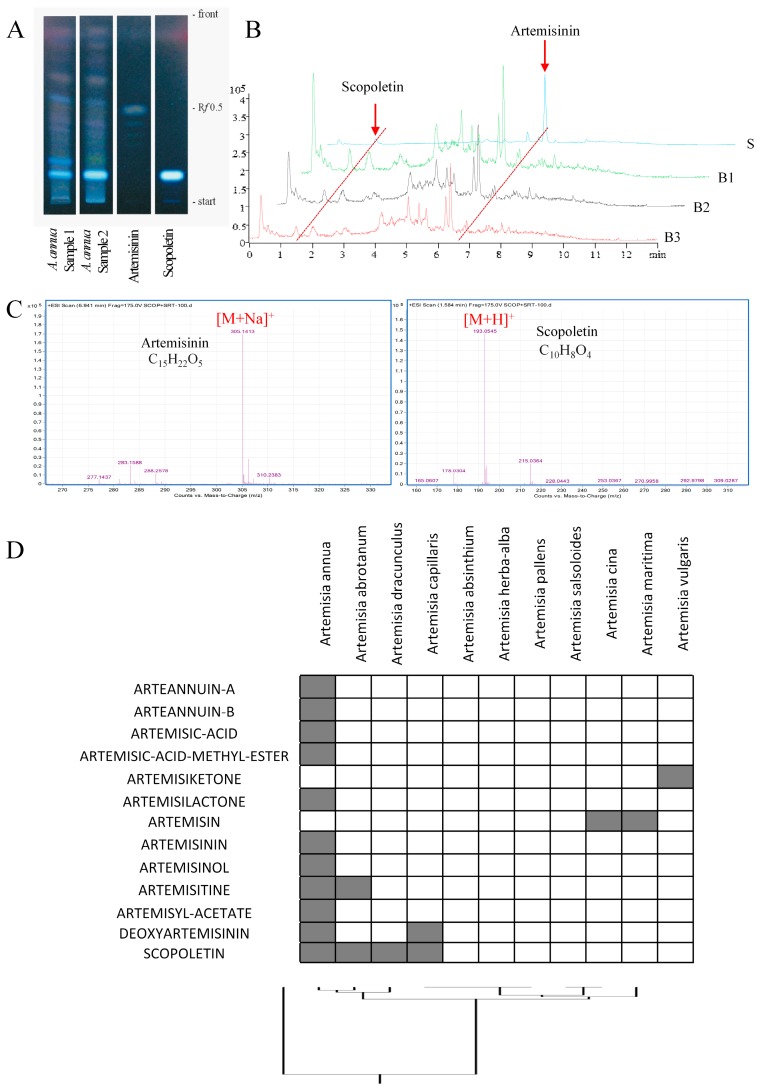
Chemoprofiling of scopoletin and artemisinin in different *Artemisia* species. (**A**) Thin layer chromatography of *Artemisia annua Herba*. Scopoletin and artemisinin were used as reference compounds. Shown are two commercial samples of *A. annua* obtained from the TCM-Hospital Bad Kötzting (Germany) of the years 1999 and 2000 (obtained with written permission of Prof. Hildebert Wagner, Ludwig-Maximilian-University Munich, Germany); (**B**) The TIC of the standard solution and three different batches of *A. annua* methanol extract. S: standard solution containing scopoletin and artemisinin; B1, B2, B3: three different batches of *A. annua* methanol extract; (**C**) Representative mass spectrum of scopoletin and artemisinin. All samples were analyzed by UHPLC-MS-TOF on an Agilent Zorbax Eclipse Plus C-18 50 mm × 2.1 mm column (particle size: 1.8 μm) at a flow rate of 0.35 mL/min. The data were acquired in the scan mode from *m*/*z* 100 to 1700 Da with 2.0 spectra/s; (**D**) Dendrogram obtained by hierarchical cluster analysis of phytochemical constituents of different *Artemisia* species. The constituents of these plants have been deposited in Dr. Duke’s Phytochemical and Ethnobotanical Databases [19,20].

As a first step, we were interested in determining the amount of scopoletin compared to artemisinin in *A. annua* and other *Artemisia* species. Thin layer chromatography demonstrated that artemisinin was only a minor constituent and scopoletin was the most abundant compound in two independent *A. annua* samples (Figure 1A).

Furthermore, we investigated the scopoletin content in three different *A. annua* methanol extract batches by UHPLC. The chromatograms, shown in Figure 1B,C, demonstrate that the composition of the three batches was stable. Artemisinin and scopoletin, with MS values of 305.1413 and 193.0545 and retention times of 6.941 and 1.584 min, respectively, have the highest abundance. According to the area and concentration of the standard compounds, the concentrations of artemisinin and scopoletin in *A. annua* methanol extract were 6.09 and 106.32 µM, respectively, suggesting that scopoletin was much more abundant in *A. annua* as compared to artemisinin.

Then, we attempted to establish chemoprofiles for 11 Artemisia species (*A. annua*, *A. abrotanum*, *A. dracunculus*, *A. capillaris*, *A. absinthium*, *A. herba-alba*, *A. pallens*, *A. salsoloides*, *A. cina*, *A. maritima* and *A. vulgaris*). We subjected the constituents of these species (Dr. Duke’s Phytochemical and Ethnobotanical Databases [19,20] to hierarchical cluster analysis (Figure 1D).

Twelve artemisinin-based compounds and scopoletin have been included in the analysis. None of the compounds were commonly found in all 11 *Artemisia* species, only scopoletin was present in four species and these species clustered together (*A. annua*, *A. abrotanum*, *A. dracunulus*, and *A. capillaris*). Artemisinin derivatives were mainly found in *A. annua*. This dendrogram demonstrates that the considerable divergence of chemical composition in the *Artemisia* species.

### 2.2. Cross-Resistance of Scopoletin to Established Anticancer Drugs

**Figure 2:**
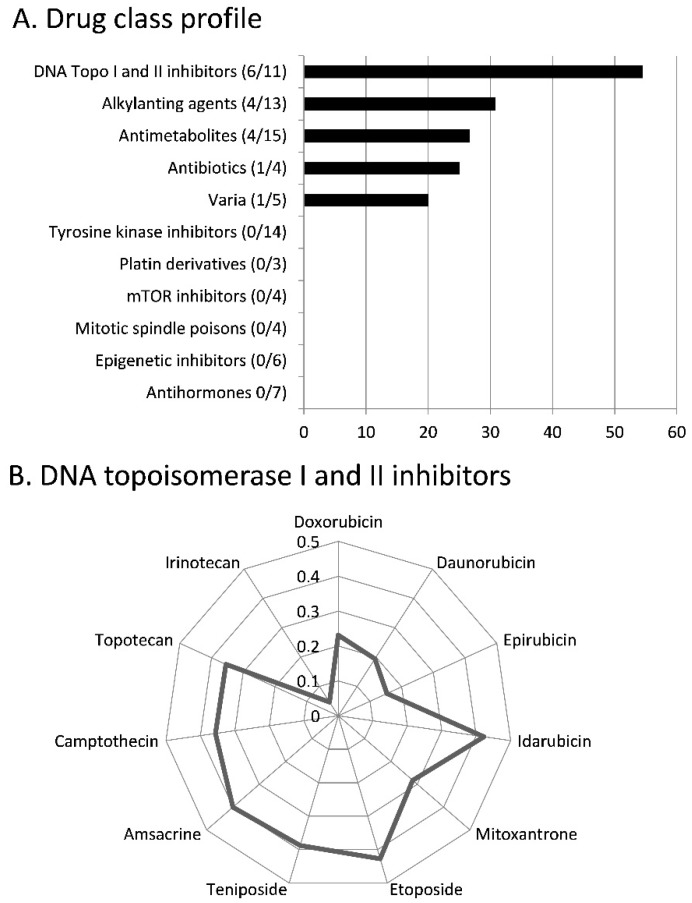
Oncobiograms for scopoletin using the NCI cell line panel. (**A**) Percentage of classes of established anticancer drugs, whose log_10_IC_50_ values correlate with those for scopoletin; (**B**) Profile of correlation values (R) DNA topoisomerase I and II inhibitors with scopoletin. The log_10_IC_50_ values are deposited at the NCI database (http://dtp.nci.nih.gov). The calculations were performed using the Pearson correlation test with *p* < 0.05 and R > 0.3 as threshold.

We correlated the log_10_IC_50_ values of the NCI cell lines to scopoletin with those of 86 standard drugs. The cellular responses of six out of 11 DNA topoisomerase I or II inhibitors drugs significantly correlated with those of scopoletin (=54.5%). Alkylating drugs were also frequently correlated to scopoletin. Four of 13 alkylating agents (=30.8%) revealed significant correlations to reserpine (*p* < 0.05; R > 0.30). Comparable results were obtained for antimetabolites (4/15 drugs = 26.7%). Intermediate correlation rates were observed for antibiotics (1/4 drugs = 25%) and various other drugs (1/15 = 20%). No correlations were found for platin compounds, tubulin inhibitors, antihormones, tyrosine kinase inhibitors, mTOR inhibitors and epigenetic inhibitors (Figure 2). The correlations to topoisomerase inhibitors, alkylating agents and antimetabolites indicate that scopoletin may interact with the DNA of tumor cells.

### 2.3. Tumor-Type Dependent Response towards Scopoletin

**Figure 3:**
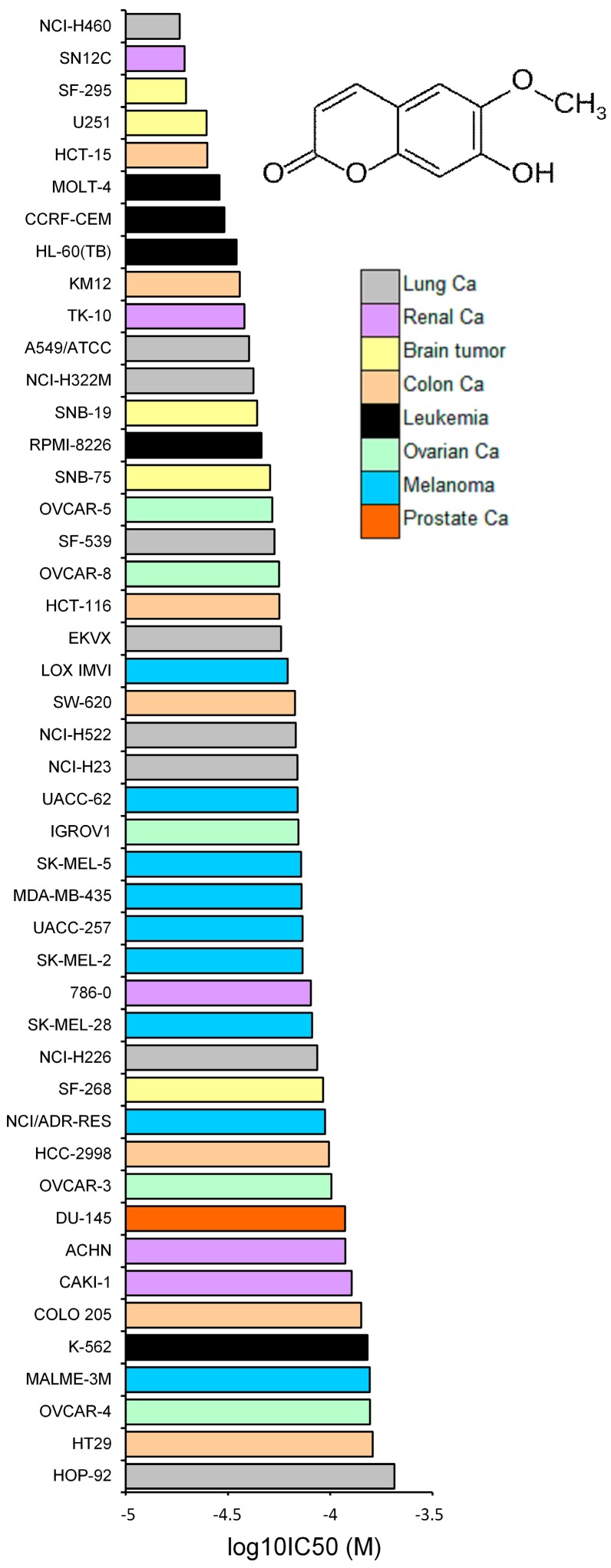
Chemical structure of scopoletin and mean values and standard deviations of log_10_IC_50_ values for scopoletin of NCI cell lines of different tumor types.

The log_10_IC_50_ values for scopoletin of all cell lines are shown in Figure 3. There was a trend whereby leukemia cell lines were more sensitive to scopoletin than cell lines from other tumor types. This result corresponds to the fact that clinically established anticancer drugs frequently show high sensitivity towards leukemia, while solid cancers tend to be more resistant to cytostatic drugs. However, among the cell lines, we did not observe any significant trend regarding tumor types, which were more sensitive and resistant to scopoletin. As the tumor type seems not to reliably predict responsiveness to scopoletin, the individual gene expression might better determine sensitivity or resistance to this compound. To analyze this hypothesis in more detail, we investigated well-known mechanisms of anticancer drug resistance, e.g., ABC-transporter genes (*ABCB1*, *ABCB5*, *ABCC1*, *ABCG2*), oncogenes and tumor suppressor genes (*EGFR*, *RAS*, *TP53*).

### 2.4. Role of Classical Drug Resistance Mechanisms for Scopoletin

ABC-transporters are well known to efflux a plethora of anticancer drugs and xenobiotic compounds protecting cancer cells from death. The clinical relevance of the major ABC-transporters ABCB1, ABCC1, and ABCG2 for failure of chemotherapy and poor survival prognosis of cancer patients has been shown [18]. ABCB5 represents a novel resistance-mediating ABC-transporter, which is expressed in cancer stem-like cells. Therefore, novel anticancer drugs are required that kill ABC-transporter-expressing MDR tumors. As shown in Table 1, the expression of none of the ABC transporters examined in our study correlated with the log_10_IC_50_ values of the NCI cell line panel for scopoletin. For each of the transporters, suitable positive control drugs have been used, which are described in the literature as substrates of the corresponding transporters [21,22,23,24]. All of the control drugs significantly correlated with expression of the corresponding ABC transporters (Table 1).

**Figure 4:**
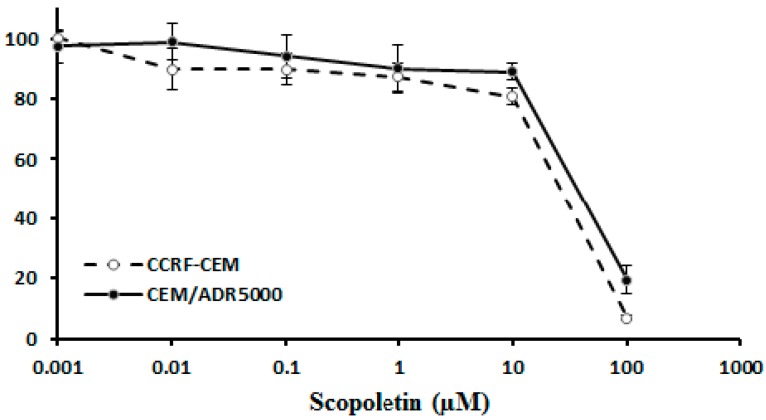
Dose response curves of scopoletin in drug-sensitive CCRF-CEM and P-glycoprotein-expressing multidrug-resistant CEM/ADR5000 cells as determined by the resazurin assay. The dose response curves show mean values ± SD of three independent experiments with each six parallel measurements.

To exemplarily confirm the results obtained by the NCI cell line panel, we tested the cytotoxicity of scopoletin in a pair of sensitive CCRF-CEM and P-gp-expressing CEM/ADR5000 cell lines using the resazurin assay. As shown in Figure 4, both cell lines were inhibited by scopletin with similar efficiency. The IC_50_ value for CCRF-CEM cells was 26.1 ± 1.6 µM and for CEM/ADR5000 was 37.5 ± 2.9 µM. The degree of resistance of CEM/ADR5000 cells to scopoletin was only 1.4, although this multidrug-resistant cell line exerts high degrees of resistance to many established anticancer drugs with degrees of resistance that are two to three orders of magnitudes higher [25]. This indicates that scopoletin is not a substrate of P-gp, which supports the results obtained by microarray analyses and correlation to log_10_IC_50_ values for scopoletin in the NCI cell line panel (Table 1).

**Figure 5:**
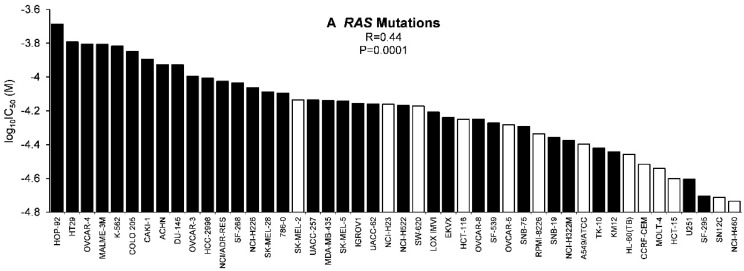

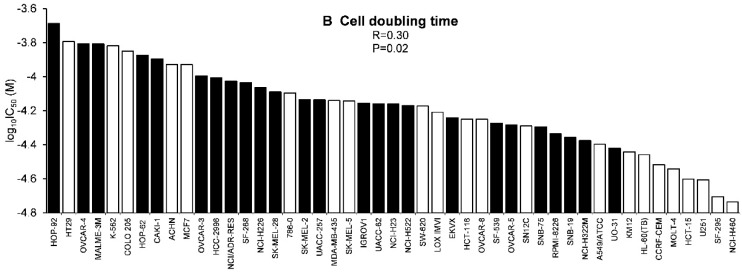
Ranked order of log_10_IC_50_ values for scopoletin of NCI cell lines in comparison to the mutational status of (**A**) pan-*RAS* (*H-*, *K-*, *N-RAS*) (solid bars mutated *RAS*, open bars wild-type *RAS*) and (**B**) cell doubling times (solid bars: slowly growing cell lines with doubling times >30 h. Open bars: rapidly growing cells with doubling times <30 h). Significance levels were calculated using two-sided Mann-Whitney’s U-test.

As tumor suppressors and activated oncogenes also contribute to drug and radiation resistance [26,27,28], we also tested whether mutations in the tumor suppressor gene *TP53* or the *H-RAS*, *K–RAS*, and *N-RAS* genes as well as the mRNA expression of the epidermal growth factor receptor gene (*EGFR*) are associated with cellular response to scopoletin. It is pleasing that we did not find significant correlations between cellular responsiveness to *EGFR* expression or *TP53* mutations (Table 1). However, the mutational status of *RAS* genes was significantly associated with cellular resistance to scopoletin, indicating that RAS may represent a resistance mechanism for scopoletin (Figure 5A).

It is well known that slowly growing tumors are more resistant to chemotherapy than fast growing ones [29]. We found that this is also true for scopoletin: the cell doubling times of the tumor cell lines were significantly correlated to the log_10_IC_50_ values for scopoletin (Figure 5B).

### 2.5. COMPARE and Hierarchical Cluster Analyses of mRNA Microarray Data

In a hypothesis-generating bioinformatic approach, we mined the NCI database and correlated the microarray-based transcriptome-wide mRNA expression of tumor cell lines with the log_10_IC_50_ values, in order to identify novel putative molecular determinants of cellular response to scopoletin. Based on Pearson’s rank correlation tests, scale rankings of genes were obtained by COMPARE analysis. The top 20 genes with positive and the top 20 with negative correlations are shown in Table 2. The identified genes belong to different functional groups such as cell cycle and proliferation (*CCNB1*, *CDKN3*, *KIF11*, *MAPRE2*, *MKI67*, *SMC3*), signal transduction processes (*ARRB2*, *ARHGAP12*, *CDC42BPA*, *MAPRE2*, *RHEB*, *TSC22D4*) and others.

**Figure 6:**
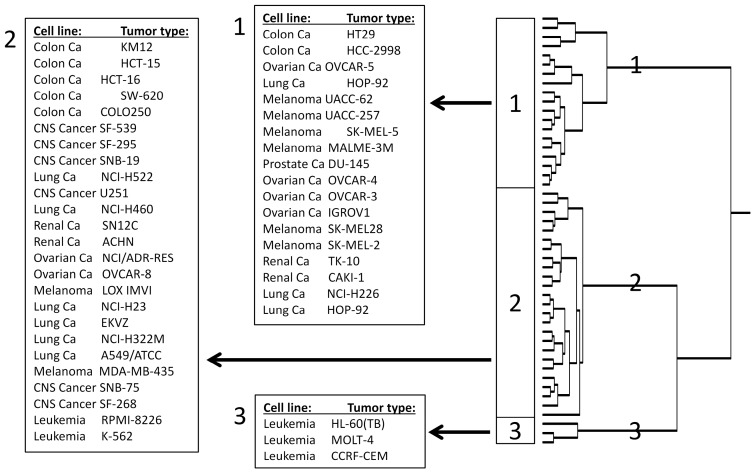
Cluster image mapping of mRNA expression of genes obtained by COMPARE analyses obtained for scopoletin. The gene expression has been determined by using the Novartis microarray platform. The dendrograms show the clustering of the NCI cell line panel according to the degrees of relatedness between cell lines. The cluster image map is based calculations with the WARD method.

Then, these genes were subjected to hierarchical cluster analysis. Only the mRNA expression data, but not the log_10_IC_50_ values of scopoletin for the cell lines were included into the cluster analysis. Three main cluster branches appeared (Figure 6). As the log_10_IC_50_ values of scopoletin were previously included into the cluster analysis, we now analyzed whether or not the obtained gene expression profile predicted sensitivity or resistance of cell lines to scopoletin. Indeed, a significant relationship was obtained (*p* = 2.05 × 10^−5^; Table 3).

### 2.6. NF-κB Motif Analysis

We hypothesized that the expression profile identified by COMPARE analysis shown in Table 2 might be commonly regulated at the transcriptional level. The idea was that a wide array of functionally diverse genes might be commonly up-or down-regulated by a transcription factor that is involved in resistance to cytotoxic compounds. We screened the gene promoter regions of this set of genes for binding motifs of the transcription factor, NF-κB to investigate this hypothesis. NF-κB mediates resistance towards diverse cancer therapeutics by inhibition of apoptosis. This broad spectrum of resistances represents another multiple drug resistance profile, which is different from the one mediated by ABC transporters, oncogenes and tumor suppressors.

**Figure 7:**
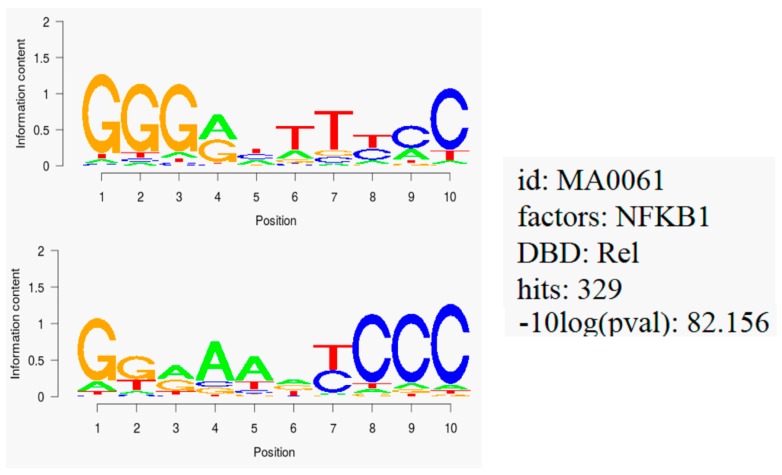
Motif analysis of 25 kb upstream regions of 40 genes identified by COMPARE analysis revealing the significant presence of NF-κB binding motif.

Interestingly, all 40 genes identified by COMPARE analysis (Table 2) harbored binding motifs for NF-κB. We performed motif analysis for upstream promoter sequences of 40 genes that correlated with the cytotoxic activity of scopoletin. With 329 hits, the NF-κB DNA binding motif (Rel) was observed to be significantly enriched (with a log *p*-value of −8.2) in regions of 25 kb upstream promoter of all genes (Figure 7), indicating that NF-κB may play a considerable role for the regulation of genes associated with cellular response to scopoletin.

### 2.7. Molecular Docking

**Figure 8:**
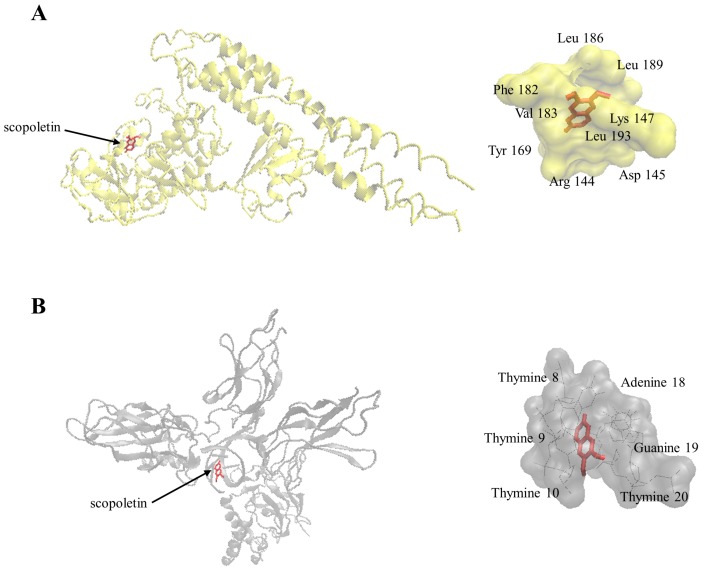
Molecular docking studies of scopoletin to NF-κB pathway proteins. (**A**) Docking pose into the ATP binding site of IΚK (PDB code: 3RZF in yellow cartoon representation); (**B**) Docking pose into the DNA binding site of NF-κB-DNA complex (PDB code: 1VKX in gray cartoon representation).

As a next step to investigate the interaction of scopoletin with the NF-κB system in more detail, we performed molecular docking analyses to study binding to NF-κB, I-κB kinase β, I-κB kinase β-NEMO complex and NF-κB-DNA complex *in silico*. Our results indeed showed that scopoletin strongly bound to the pharmacophores of two of these target proteins. Scopoletin bound with calculated binding energies of less than −7 kcal/mol to the NF-κB DNA complex and I-κB kinase (Figure 8 and Table 4), but not to free NF-κB and the I-κB kinase-NEMO complex (data not shown). Hence, scopoletin may target different steps of the NF-κB pathway to modulate NF-κB-mediated functions.

### 2.8. NF-κB Reporter Assay

**Figure 9:**
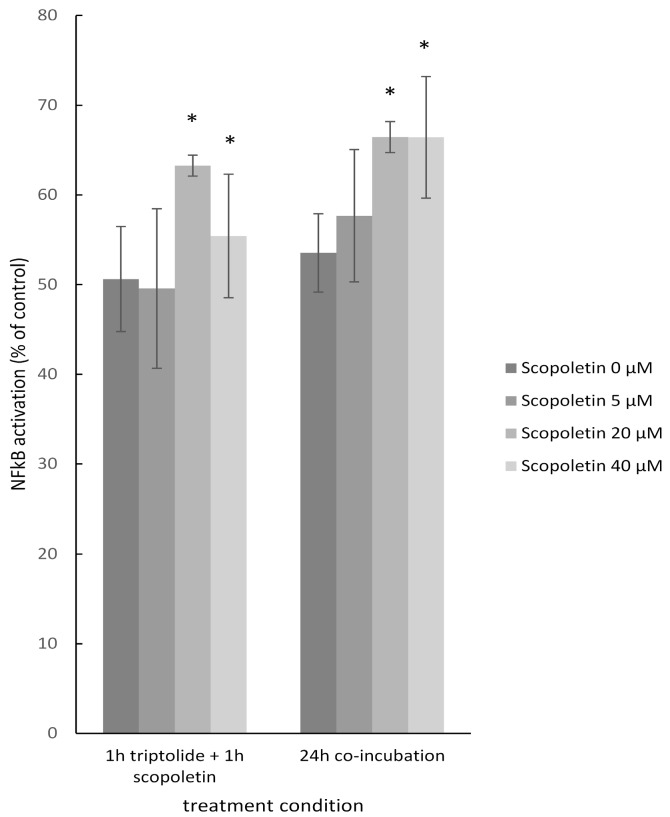
Effect of triptolide and scopoletin on NF-κB activity. 1.6 nM triptolide and various concentrations of scopoletion (0 µM, 5 µM, 20 µM or 40 µM) were treated in two different conditions; 1 h triptolide treatment and then 1 h scopoletin treatment, 24 h co-incubation of triptolide and scopoletin. Quantification was carried out according to the untreated control and experiments were performed three times. (Significantly different according to Student’s *t*-test, * *p*-value < 0.05).

The effects of scopoletin on NF-κB are controversially discussed in the literature. This compound can either activate or inhibit the activity of this transcription factor [32,33]. From our molecular docking studies we obtained only clues for binding, which implies an interaction of scopoletin with the transcription factor machinery. However, it cannot be concluded, whether this interaction leads to activation or inhibition. Therefore, we used a SEAP-driven NF-κB reporter cell line to address this question. Preliminary experiments with this cell line showed that scopoletin did not inhibit TNF-α-induced NF-κB activation. Therefore, we investigated, whether scopoletin would rather activate NF-κB. For this reason, we partially inhibited NF-κB by a low concentration (1.6 nM) of a well-known NF-κB inhibitor, triptolide [34,35]. Indeed, scopoletin (20 µM or 40 µM) significantly induced NF-κB activity (Figure 9). Activation of NF-κB by scopoletin was observed at two different treated conditions: incubation with triptolide for 1 h followed by incubation with scopoletin for another hour and co-incubation with both triptolide and scopoletin for 24 h (Figure 9).

## 3. Discussion

### 3.1. Chemoprofiling

As a part of our ongoing research on the anticancer activity of *A. annua*, in the present study we focused on scopoletin. While artemisinin-based compounds are mainly found in *A. annua* alone, scopoletin can be found in other *Artemisia* species and also in other genera (*Brunfelsia*, *Kleinhovia*, *Mallotus*, *Scopolia*, *Solanum*, *Urticaria*, *Viburnum*). Although the use of chemoprofiles has been proposed as a taxonomical parameter, our results with 11 different *Artemisia* species do not support this concept. This observation is in accordance with data obtained for other plant genera [36].

Artemisinin is the most well-known constituent of *A. annua* and it is overseen sometimes that other bioactive compounds are also present in the plant [37]. In two independent laboratories in Germany and China, two samples analyzed by TLC and another three samples by UHPLC showed that scopoletin was much more abundant than artemisinin in *A. annua*. Previously, other authors also reported that *A. annua* contains scopoletin [38,39]. In addition to scopoletin and artemisinin, it is well possible that some more phytochemicals of *A. annua* reveal cytotoxicity towards cancer cells [15]. *A. annua* is frequently used in Africa outside the approved clinical practice by indigenous people as herbal tea to treat malaria. Although hot water decoctions indeed show some activity against malaria, WHO warns against the use of herbal teas instead of approved artemisinin combination therapies (ACT). The apprehension is that suboptimal artemisinin concentrations in the tea may or may not treat malaria on the short run, but would foster the development resistance on the long run. Of course, that would be a fatal consequence that has to be avoided. However, several aspects have to be considered in this context.

Artemisinin is a rather water insoluble molecule and it cannot be assumed that herbal teas contain considerable amounts of artemisinin. The observed anti-malaria effects of *A. annua* decoctions might be due to other water-soluble compounds in the plant. The problem of poor water-solubility of artemisinin is known since the detection of artemisinin. Youyou Tu, who obtained the Nobel Price for Physiology or Medicine 2015, obtained inconsistent antimalarial effects at the beginning. Only as she went back to the original historical records on the traditional use in Chinese medicine (Handbook of Prescriptions for Emergency Treatment, *Hou Bei Ji Fang*, by Hong Ge, 281–340 B.C.), she recognized that the press juice rather than hot water decoction was recommended [8]. Furthermore, plant extracts prepared by water decoction, press juice or other forms contain always more than one single phytochemical and are combination therapies. Hence, plant extracts act against the development of resistance, since different compounds reveal different modes of action. Indeed, this has recently been shown in animal experiments [40]. Whole plant preparations of *A. annua* overcame existing resistance to pure artemisinin in the rodent malaria *Plasmodium yoelii*. In a long-term artificial selection for resistance in *Plasmodium chabaudi* and stable resistance to *A. annua* whole plant preparations was achieved three times more slowly than stable resistance to pure artemisinin. The authors concluded that the resilience of the whole plant may be attributable to a multicomponent defense system, which is more effective than isolated single compounds.

### 3.2. Cross-resistance and Drug Resistance Mechanisms

The correlation analysis of scopoletin with a panel of standard anticancer agents revealed that scopoletin tends to be cross-resistant to drugs targeting DNA, e.g., DNA toposiomerase I or II inhibitors, alkylatin agents, or antimetabolites, but not to mitotic spindle poisons or antihormones. Given that scopoletin might be developed as anticancer drug in the future, combination therapy with classical DNA-damaging anticancer drugs might not lead to improved tumor cell killing because of resistance to drugs affecting tumor DNA. It is interesting that not only cellular response to scopoletin was not associated with anticancer drugs addressing other cellular targets, but also that some of them belong to novel categories of targeted chemotherapeutics such as tyrosine kinase inhibitors, mTOR inhibitors or epigenetic drugs. This specific cross-resistance profiles that spares these targeted drugs may open a therapeutic window for novel combination therapies of scopoletin. The fact that scopoletin derivatives have been developed that are active against human xenograft tumors *in vivo* [41,42] can be taken as a clue that this natural product may be subjected to drug development to find its way into the clinic.

A central topic of our study was to investigate whether scopoletin may be active against otherwise drug-resistant tumor cells. Because of resistance to a broad spectrum of anticancer drugs [28], novel drugs with improved features are desperately required in clinical oncology. Out of the 48 human ABC-transporters, P-gp/*MDR1/ABCB1*, *ABCB5*, MRP1/*ABCC1* and BCRP/*ABCG2* have been extensively shown to be associated with MDR phenomena in human tumors [18]. Therefore, we analyzed the role of these ABC transporters to scopoletin. We found that the expression and/or function of these efflux pumps were not related to the cytotoxic activity of scopoletin, indicating that MDR caused by these ABC transporters may not hamper the cellular responsiveness towards scopoletin. It has been recently shown that P-glycoprotein in the blood-brain barrier did not affect scopoletin transport [40], a result which is in agreement with our data. The role of other ABC transporters for cytotoxicity of scopoletin against cancer cells has not been investigated.

We also found that the expression of *EGFR* and mutation of *TP53* did not affect scopoletin’s activity in cancer cells, while mutations in *RAS* oncogenes were significantly correlated with scopoletin resistance. *EGFR* represents an important oncogene that triggers malignant growth [43,44]. *EGFR*-overexpression is associated with unfavorable response to chemotherapy and short survival times of cancer patients. Mutations in the *EGFR* gene confer drug resistance. Again, scopoletin inhibited cancer cell lines independent of their *EGFR* expression, indicating that this oncogene does not represent a resistance factor for scopoletin. These results fit to data published recently, which showed that *Morinda citrifolia* extract rich in scopoletin (and epicatechin) inhibited lung tumor growth *in vivo* and down-regulated EGFR and other tumor markers [45].

*RAS* mutations are known for quite a long time to cause resistance to DNA-damaging drugs and irradiation [46,47]. We found that resistance to scopoletin was also associated with the mutational status of *RAS* oncogenes. This result fits to the correlation of scopoletin’s cellular responsiveness to those of DNA-damaging agents (see above), which supports the assumption that scopoletin might also act against tumor cells by damaging DNA.

There is a long-lasting debate in the literature about the clinical relevance of the tumor suppressor *TP53* for therapy against radioresistance [27]. DNA lesions are recognized by p53 leading to cell cycle arrest and DNA repair. If damage exceeds the cellular repair capability, *TP53* may sense the induction of apoptosis. Both DNA repair and apoptosis ultimately maintain health of the organism. *TP53* mutations lead to p53 protein which lost its function. This in turn causes carcinogenesis and drug resistance. It was pleasing to observe that scopoletin activity is not related to the *TP53* mutational status, indicating that *TP53*-mutated otherwise resistant tumors may still be responsive to scopoletin.

### 3.3. COMPARE and Hierarchical Cluster Analyses of mRNA Microarray Data

Many—if not all—natural products act in a multifactorial manner [41,42,44,45,48,49,50,51,52,53,54]. Therefore, we performed COMPARE and hierarchical cluster analyses of transcriptome-wide microarray-based mRNA expressions. We identified a gene expression profile, which was significantly correlated with the log_10_IC_50_ values of the NCI cell lines for scopoletin. Genes of diverse functional groups and signaling routes appeared. The identified genes belong to many different functional groups, although genes regulating the cell cycle and cell proliferation (*CCNB1*, *CDKN3*, *KIF11*, *MAPRE2*, *MKI67*, *SMC3*) as well as genes involved in signal transduction (*ARRB2*, *ARHGAP12*, *CDC42BPA*, *MAPRE2*, *RHEB*, *TSC22D4*) appeared more frequently than genes from other functional classes. This may indicate that these two cellular processes may be of specific relevance for the responsiveness of tumor cells towards scopoletin. Although the role of the specific genes identified here for cellular responsiveness towards scopoletin is still unknown, cell cycle regulating genes [46,47,49,50] and signal transduction pathways in general play a tremendous role for cancer drug resistance [51,52,53]. Therefore, genes involved in cell cycle and proliferation as well as signal transducing processes may contribute to sensitivity or resistance of tumor cells to scopoletin.

### 3.4. NF-κB as Mechanism of Therapy Resistance

NF-κB mediates resistance towards diverse cancer therapeutics by inhibition of apoptosis, and inhibition of NF-κB sensitizes cancer cells towards anticancer drugs (e.g., doxorubicin, imatinib), cytotoxic phytochemicals (e.g., curcumin), biological agents (e.g., β-IFN, TRAIL), and radiation [47,54,55,56,57,58,59]. This broad spectrum of resistances represents another multiple drug resistance profile, which is different from the one mediated by ABC transporters, oncogenes and tumor suppressors.

The fact that all 40 genes identified by COMPARE analysis harbored binding motifs in their gene promoter sequences and that scopoletin binds *in silico* to the NF-κB-DNA complex as well as the ATP binding site of IΚK speaks for a role of NF-κB in regulating cellular response to scopoletin. By using a SEAP-driven NF-κB reporter cell line, we found that scopoletin activated NF-κB. We suggest that NF-κB activation might confer drug resistance. This implies that NF-κB may be a resistance mechanism of cancer cells towards scopoletin and other drugs, although main resistance mechanisms such as ABC-transporters, *EGFR*, and *TP53* do not affect cellular resistance to scopoletin.

### 3.5. Mutagenic and Genotoxic Potential of Scopletin

Scopoletin as well as 8-methoxypsoralen (8-MOP) are coumarin derivatives. 8-MOP is an approved drug, which is used together with UV-A light for photochemotherapy (PUVA therapy) of vitilago and for photophoresis of Sezary syndrome. There is controversy about the long-term cancer risk of PUVA therapy [60,61]. Therefore, the mutagenic and genotoxic potential of coumarin has been investigated, since coumarin is present in foods and used in cosmetic fragrances. The majority of investigations suggest that coumarin is not a genotoxic agent [62]. The contrasting data in animal and human experimental models may be explained by different metabolization routes. In human beings the prevalent detoxification pathway is the 7-hydroxylation pathway, whereas the 3,4-epoxidation pathway predominant in rats and mice leads to the formation of toxic, harmful metabolites [63]. Hence, coumarin in food and cosmetics bears no health risk for human subjects. The mutagenic potential of scopoletin is not well investigated as of yet. Scopoletin can even act in an anti-mutagenic manner by inhibiting the mutagenicity of cigarette smoke condensate [64]. Further safety assessment is necessary, if scopoletin would be further developed as anticancer drug.

The prospect that scopoletin might serve as lead compound for drug development and would find its way into the clinics, is supported by the favorable activity against tumors expressing well-known drug resistance mechanisms, although *RAS* mutations and NF-κB may hamper the effectiveness of scopoletin. Further investigations are warranted to explore the full therapeutic potential of this natural product.

## 4. Material and Methods

### 4.1. Chemicals and Extracts

*Artemisia* samples were harvested in the years 1999 and 2000 and kindly provided by the TCM Hospital Bad Kötzting (Bad Kötzting, Germany). Artemisinin and scopoletin were used as standards. Artemisinin was isolated from *A. annua*, whereas scopoletin was obtained from Sigma-Aldrich (Taufkirchen, Germany). Three different batches of *A. annua* were purchased from Anguo Ruiqi Chinese Herbal Medicines Co., Ltd. (Anguo, China).

### 4.2. Thin Layer Chromatography

The thin layer chromatopgraphy (TLC) method was performed as described [64].

### 4.3. Preparation of Standard Solutions and the A. annua Methanol Extract

Artemisinin and scopoletin standard solutions were prepared by dissolving them with DMSO to a concentration of 50 mM, then diluted into 50 µM with methanol, and 1 μL of 50 µM standard solution was injected for analysis. The methanol extracts of *A. annua* was prepared as follows: 5 g *A. annua* powder was refluxed with 200 mL methanol for 1 h. Then, this procedure was repeated with 150 mL methanol for another 1 h. The solution was set to 350 mL, 1 mL of the extraction solution was filtered and 1μL was injected for analysis.

### 4.4. Ultra-High Pressure Liquid Chromatography (UHPLC)

UHPLC (1290 Series, Agilent Technologies, Santa Clara, CA, USA) equipped with time of flight mass spectrometry (TOF-MS; Agilent Technologies 6230) with a jet stream ion source was operated in negative ion mode during the UHPLC analyses. The samples were analyzed by using an Agilent Zorbax Eclipse Plus C-18 column (50 mm × 2.1 mm) with a particle size of 1.8 μm (flow rate: 0.35 mL/min). The parameters of the gradient elution program were applied as follows: mobile phase A (0.1% formic acid in water) and mobile phase B (0.1% formic acid in ACN): 0–3 min, 20% (B); 2–8 min, 20%–80% (B); 8–9 min, 80%–100%; 9–10 min, 100%–20% (B); 10–13 min, 20% (B). For UHPLC/TOF-MS analyses, the data were acquired in the scan mode (*m/z* 100 to 1700 Da with 2.0 spectra/s). Data were analyzed by using Agilent MassHunter Workstation software B.01.03.

### 4.5. Cell Lines

A panel of 56 human tumor cell lines of the Developmental Therapeutics Program of the NCI (Bethesda, MD, USA) consisted of leukemia, melanoma, non-small cell lung cancer, colon cancer, renal cancer, ovarian cancer, breast cancer, and prostate carcinoma cells as well as tumor cells of the central nervous system [65]. Cells treated with scopoletin for 48 h were assayed by means of a sulforhodamine B assay [66].

### 4.6. Resazurin Assay

The cytotoxicity of scopletin has been assayed by resazurin reduction [24]. Resazurin as indicator dye is reduced to highly fluorescent resorufinin viable cells, but not in dead cells, which cannot metabolize resazurin anymore. We previously described the protocol for the resazurin assay and the cell lines (drug-sensitive CCRF-CEM leukemia cells and their P-gp-expressing multidrug-resistant CEM/ADR5000 subline) in detail [26,27]. The cells were treated with scopoletin for 72 h. Experiments were performed three times with at least six replicates per experiment. The 50% inhibition concentrations (IC_50_) were calculated from dose response curves of each cell using Excel 2013 software (Microsoft, Redmond, WA, USA).

### 4.7. DNA Analyses with the NCI Cell Line Panel

DNA analyses focused on the measurement of gene copy numbers and point mutations. Gene copy numbers relative to normal female DNA were determined using array comparative genomic hybridization (*ABCB1*, *ABCC1*, *EGFR*). DNA samples were labeled with Cy3-dUTP and Cy5-dUTP were hybridized to OncoBAC DNA microarrays and quantitated. Normal human female DNA was used for the control.

Point mutations in the *TP53* gene were detected by cDNA sequencing. PolyA mRNA was extracted from cells using a MicroFast Track mRNA isolation kit (Invitrogen, Waltham, MA, USA) and cDNA was prepared using a First-Strand cDNA synthesis kit (Pharmacia, Berlin, Germany) according to the manufacturer’s recommendations. Three overlapping *TP53*-specific PCR primers covering the entire open-reading frame were used to bi-directionally sequence the *TP53* cDNA using Taq dideoxy-sequencing methodology on an Applied Biosystems model 373A automated sequencer (Applied Biosystems, Waltham, MA, USA).

The transcriptional activity of *TP53* cDNA from individual cell lines was measured in a yeast survival assay. The assay uses 3 centromeric plasmids. The 1st plasmid, pLS76 (positive control), has the *LEU2* gene as a selectable marker and also full length wild-type human *TP53* cDNA under the control of the *ADH1* promoter. The 2nd plasmid, pSS16, is identical to pLS76 except that the wild-type *TP53* sequence from codons 68 through to 347 is replaced by the selectable marker *URA3*. The 3rd plasmid, pSS1, contains a *HIS3* gene under the control of a single *TP53* binding site derived from the ribosomal gene cluster. Input *TP53* was generated from polyA mRNA extracted from cells. *TP53* cDNA was PCR amplified using Pfu polymerase (Stratagene, La Jolla, CA, USA) and yeast were co-transformed with the PCR generated *TP53* and HindIII/StuI restricted plasmid pSS16 (which releases the *URA3* gene segment). Repair of the gapped pSS16 plasmid with the *TP53* derived from the input *TP53* PCR product occurs *in vivo* through homologous recombination. Transformants which have successfully repaired pSS16 were selected on media lacking leucine and such replicates lack growth on uracil-minus media. If the yeast has been also transfected with pSS1 then growth on media lacking histidine identifies colonies that contain transcriptionally active *TP53*. Leu+ colonies containing only wild-type *TP53* sequence grew successfully on plates lacking histidine. Leu+ colonies containing only *TP53* loss-of-function mutations failed to grow on plates lacking histidine. Leu+ colonies derived from *TP53* cDNA prepared from cancer cells containing heterozygous *TP53* gene status yield approximately 50% His+ colonies.

To determine the mutational status in *H-*, *K-* and *N-RAS* genes, Genomic DNAs were isolated using a Cell Culture DNA kit (Qiagen, Hilden, Germany). Exons 1 and 2 of each RAS gene were amplified by PCR from the genomic DNA’s using primer pairs specific for each exan/intron junction. PCR amplifications were performed in a GeneAmp PCR System 9600 (Perkin-Elmer, Rodgau, Germany) and were all preceded with one cycle of denaturation at 94 °C for 3 min and finished with the incubation at 72 °C for 3 min. The PCR conditions used were 40 cycles of 94 °C (15 s), 48 °C (15 s), 72 °C (15 s with 1 s extension per cycle) for *K-RAS2* exon 1 and *N-RAS* exon 1; 94 °C (15 s), 52 °C (15 s), 72 °C (15 s with 1 s extension per cycle) for *K-RAS2* exon 2 and *H-RAS1* exon 2; 94 °C (15 s), 48 °C (15 s), 72 °C (25 s with 1 s extension per cycle) for *N-RAS* exon 2 and 94 °C (15 s), 56 °C (15 s), 72 °C (15 s with 1 s extension per cycle) for *H-RAS1* exon 1. Products were purified from a 6% polyacrylamide gel. DNA sequence was determined for both strands directly from the purified PCR products using the same primers as those for PCR amplification. DNA sequencing reactions were carried out with the PRISM Ready Reaction DyeDeoxy Terminator cycle sequencing kit (Applied Biosystems, Waltham, MA, USA), and the products were analyzed on an ABI 373A DNA sequencer.

### 4.8. mRNA Analyses with the NCI Cell Line Panel

The transcriptome-wide mRNA expression measured by microarray hybridizations as well as data from diverse assays to validate the microarray results have been deposited at the website of the Developmental Therapeutics Program of the NCI (http://dtp.nci.nih.gov). The expression of *ABCB1*, *ABCB5*, and *EGFR* was measured utilizing Affymetrix Human Genome U95Av2 A-E arrays and the expression of *ABCC1* and *ABCG2* using Affymetrix U133A and U133B chips. Many of these probe sets can be linked to known genes (based on clustering information from UniGene and GeneCards, where information on possible function can be found). All gene assignments should be considered tentative. Sequence information for a probe set can be found through GenBank or UniGene.

For validation of microarray-based mRNA data, different methods have been used. For measurement of *ABCB1* or *ABCB5,* total RNA was purified with the RNeasy kit. Levels of ABC transporters were measured by real-time quantitative RT-PCR using the LightCycler RNA Amplification SYBR Green kit and a Roche LightCycler machine. The gene-specific data was normalized to actin RNA levels, and are the average of two experiments.

Messenger RNA expression of *ABCC1* was measured relative to SW620 cells. Total RNA was extracted and subjected to quantitative PCR using serially diluted samples. RNA isolated from SW620 was used as a reference standard. The level in the SW 620 cell line was arbitrarily assigned a value of 10, and all other values were determined relative to this. All quantifications were performed by densitometry.

Expression of *EGFR* mRNA expression was determined by RNAse protection assay. A 141bp fragment of *EGFR* cDNA, a 470-bp fragment of c-erbB2 cDNA, and a 603-bp fragment of *TGFA* DNA were used to generate ^32^P-labeled antisense riboprobes. Thirty milligrams of total RNA were hybridized with 3 × 10^5^ cpm-labeled riboprobe and digested for 30 min at 25 °C with RNases A and T1. Following extraction, samples were separated by PAGE and autoradiographed. The relative amount of mRNA was quantified using a Phosphor Imager.

### 4.9. Protein Analyses with the NCI Cell Line Panel

To determine the protein expression of ABCG2, western blotting was performed. Cell pellets were solubilized in a lysis buffer, resolved by SDS-PAGE, transferred onto nitrocellulose membranes and incubated with an anti-BCRP polyclonal antibody. Blots were incubated with a peroxidase-conjugated donkey anti-rabbit secondary antibody and visualized using ECL Plus chemiluminescence detection kit (GE Healthcare, Chicago, IL, USA).

The EGFR protein expression was measured using Rev Prot Lysate Array. Protein lysates were diluted to a uniform protein concentration, denatured, and dilution series printed onto slides. These arrays were probed with antibody specific for the indicated protein, using the DAKO signal amplification system.

### 4.10. Other Analyses with the NCI Cell Line Panel

#### 4.10.1. Rhodamine 123 Efflux Assay

P-glycoprotein encoded by *ABCB1* acts as drug efflux pump. Rhodamine 123 is a fluorescent substrate for P-glycoprotein, which can be used to measure the functional activity of this drug transporter. Suspensions of logarithmic phase cells were obtained from tissue culture plates by trypsinization. During the accumulation period, four aliquots of cells were re-suspended in rhodamine-containing medium (improved minimum essential medium with 10% fetal calf serum and 0.5 µg/mL rhodamine 123) and incubated with or without 3.0 µg/mL cyclosporin A at 37 °C in 5% CO_2_ for 30 min. After the accumulation period, efflux was initiated by sedimentation at 600× g and resuspension in rhodamine-free medium (improved minimum essential medium with 10% fetal calf serum), with or without 3.0 µg/mL cyclosporin A. The efflux was carried out at 37 °C in 5% CO_2_ for 90 min. At the end of both the accumulation and efflux periods, cells were sedimented, washed in ice-cold Hank’s buffered salt solution, placed in Hank’s buffered salt solution with 10% fetal calf serum on ice, and kept in the dark until flow cytometric analysis. Samples were analyzed on a FACScan flow cytometer equipped with a 488 nm argon laser. The green fluorescence of rhodamine 123 was collected after a 530 nm band pass filter. Samples were gated on forward scatter *versus* side scatter to exclude cell debris and clumps. A minimum of 6000 events were collected for each sample. Histograms were generated for each cell line and the median rhodamine fluorescence obtained for each peak were identified by channel number, and this number (denoting the logarithm of fluorescence intensity) was used to indicate the amount of rhodamine in the cells in each incubation condition. A difference of 256 channel numbers represents a 10-fold change in fluorescence intensity.

#### 4.10.2. Cell Doubling Times

The cell lines in the panel grow at different rates, which may affect sensitivity to certain compounds. Doubling time (in hours) was obtained by determining the change in SRB signal over time at the standard conditions and cell densities used for the NCI anticancer drug screen. These data are averages from 8 years of screening.

### 4.11. Statistical Analysis

Data on cytotoxicity (log_10_IC_50_ values) and mRNA microarray data of the NCI tumor cell line panel [67] are deposited at the NCI website (http://dtp.nci.nih.gov). For hierarchical cluster analysis, objects were classified into dendrograms by calculating distance according to the closeness of between-individual distances by means of the Ward method (WinSTAT program, Kalmia, Cambridge, MA, USA). Cluster models have been previously validated for gene expression profiling and for approaching molecular pharmacology of cancer [68,69]. The application of this method for pharmacogenomics of phytochemicals has been described in detail [15].

COMPARE analyses were performed to produce rank-ordered lists of genes expressed in the NCI cell lines as previously described [70,71]. Briefly, every gene of the NCI microarray database was ranked for similarity of its mRNA expression to the log_10_IC_50_ values for scopoletin. To derive COMPARE rankings, a scale index of correlation coefficients (R-values) was created.

Pearson’s correlation test was used to calculate significance values and rank correlation coefficients as a relative measure of the linear dependency of two variables (WinSTAT, Kalmia). The chi-squared test was applied to bivariate frequency distributions of pairs of nominal scaled variables (WinSTAT, Kalmia). It was used to calculate significance values (*p-*values) and rank correlation coefficients (R-values) as a relative measure of the linear dependency of two variables.

### 4.12. Binding Motif Analysis

COMPARE analysis showed 40 genes that either directly or inversely correlated with log_10_IC_50_ values of a panel of 60 NCI cell lines upon scopoletin treatment. Twenty five kilobases long upstream promoter sequences were retrieved from UCSC Genome Browser Gene Sorter (http://genome.ucsc.edu) and BED (Browser Extensible Data) files were created to screen for the possible binding motifs for each gene. Promoter sequences were screened using SeqPos tool implemented in Galaxy, Cistrome software [72].

### 4.13. Molecular Docking

Molecular docking was performed to calculate the interaction energy and geometry of scopoletin with I-κB kinase β, I-κB kinase β-NEMO (NF-κB essential modulator) complex, NF-κB, and NF-κB-DNA complex. The protocol for molecular docking was previously reported by us [33]. X-ray crystallography-based protein structures were obtained from Protein Data Bank [73,74]; I-κB kinase β (PDB ID:3RZF), I-κB kinase β-NEMO complex (PDB ID: 3BRT), NF-κB (p52/RelB heterodimer, PDB ID: 3DO7), NF-κB-DNA complex (p50/p65 heterodimer bound to DNA, PDB ID: 1VKX) and set as rigid receptor molecules. A grid box was then constructed to define docking spaces in each protein according to its pharmacophores. Docking parameters were set to 250 runs and 2,500,000 energy evaluations for each cycle. Docking was performed three times independently by Autodock4 and with AutodockTools-1.5.7rc1 [34] using the Lamarckian Algorithm. The corresponding lowest binding energies and predicted inhibition constants were obtained from the docking log files (dlg). Mean ± SD of binding energies were calculated from three independent dockings. Visual Molecular Dynamics (VMD) was used to depict the docking poses of scopoletin.

### 4.14. NF-κB Reporter Assay

HEK-Blue-Null1 was derived from the human embryonic kidney-293 (HEK-293) cell line and obtained from Invivogen (San Diego, CA, USA). These cells stably express an optimized secreted embryonic alkaline phosphatase (SEAP) reporter gene preceded by the NF-κB promoter.

In a 96-well plate, 5.0 × 10^4^ HEK-Blue-Null1 were incubated overnight at 37 °C in Dulbecco’s modified Eagle’s medium (DMEM; Gibco, Dreieich, Germany) supplemented with 2 mM l-glutamine, 10% (*v*/*v*) heat-inactivated fetal bovine serum (FBS), 50 units/mL penicillin, 50 µg/mL streptomycin (Gibco) and 100 µg/mL Normocin (Invivogen). Cell were treated with 1.6 nM triptolide (Invivogen) and varying concentrations of scopoletin (5 µM, 20 µM and 40 µM) (Sigma-Aldrich). The activation of NF-κB was induced by 100 ng/mL of TNF-α for 24 h. Then, 20 µL aliquots of cell culture were added to 180 µL of pre-warmed Quanti-Blue detection reagent (Invivogen) per well according to the manufacturer’s instructions. NF-κB activation was detected by measuring SEAP spectrophotometrically at 630 nm (Tecan Teader, Tecan Group Ltd., Maennedorf, Switzerland).

## Figures and Tables

**Table 1:** Correlation of log_10_IC_50_ values for scopoletin to ABC transporters, oncogenes and tumor suppressor genes in the NCI cell line panel. Known standard compounds were used as positive controls. The analysis was performed by means of Pearson’s rank correlation test.

		Scopoletin	Control Drug
		(log_10_ IC_50_, M)	(log_10_ IC_50_, M)
**ABCB1 Expression**			Daunorubicin
7q21 (Chromosomal	R-value	0.099	* 0.597
Locus of *ABCB1* Gene)	*p-*value	0.262	* 4.82 × 10^−6^
*ABCB1* Expression	R-value	0.071	* 0.684
(Microarray)	*p-*value	0.317	* 1.57 × 10^−8^
*ABCB1* Expression	R-value	−0.146	* 0.579
(RT-PCR)	*p-*value	0.167	* 4.19 × 10^−6^
Rhodamine 123	R-value	−0.017	* 0.544
Accumulation	*p-*value	0.453	* 1.51 × 10^−5^
**ABCB5 Expression**			Maytansine
*ABCB5* Expression	R-value	0.094	* 0.454
(Microarray)	*p-*value	0.265	* 6.67 × 10^−4^
*ABCB5* Expression	R-value	0.024	* 0.402
(RT-PCR)	*p-*value	0.438	* 0.0034
**ABCC1 Expression**			Vinblastine
DNA Gene	R-value	0.003	* 0.429
Copy Number	*p-*value	0.493	* 0.001
*ABCC1* Expression	R-value	−0.199	* 0.399
(Microarray)	*p-*value	0.090	* 0.002
*ABCC1* Expression	R-value	−0.129	0.299
(RT-PCR)	*p-*value	0.181	* 0.036
**ABCG2 Expression**			Pancratistatin
*ABCG2* Expression	R-value	−0.311	* 0.323
(Microarray)	*p-*value	0.017	* 0.006
ABCG2 Expression	R-value	−0.310	* 0.346
(Western Blot)	*p-*value	0.017	* 0.004
**EGFR Expression**			Erlotinib
*EGFR* Gene	R-value	−0.123	−0.245
Copy Number	*p-*value	0.205	* 0.029
*EGFR* Expression	R-value	−0.095	* −0.458
(Microarray)	*p-*value	0.262	* 1.15 × 10^−4^
*EGFR* Expression	R-value	0.056	* 0.409
(RNAse Protection)	*p-*value	0.358	* 7.08 × 10^−4^
EGFR Expression	R-value	−0.096	* −0.376
(Protein Array)	*p-*value	0.260	* 0.001
**TP53 Mutation**			5-Fluorouracil
*TP53* Mutation	R-value	0.070	* −0.502
(cDNA Sequencing)	*p-*value	0.323	* 3.50 × 10^−5^
TP53 Function	R-value	−0.026	* −0.436
(Yeast Functional Assay)	*p-*value	0.434	* 5.49 × 10^−4^

* *p* < 0.05 and R > 0.3 (or R < −0.3).

**Table 2:** Correlation of constitutive mRNA expression of genes identified by COMPARE analyses with log_10_IC_50_ values of scopoletin for the NCI tumor cell lines.

COMPARE Coefficent	Experimental ID	GenBank Accession	Gene Symbol	Name	Function
−0.542	GC37742	M25753	*CCNB1*	Cyclin B1	G2/M cell cycle transition
−0.496	GC31070	X70944	*SFPQ*	Splicing factor proline/glutamine-rich	DNA- and RNA binding protein
−0.476	GC32832	D29012	*PSMB6*	Proteasome (prosome, macropain) subunit, β type, 6	Proteasome component
−0.472	GC27340	U16954	*MLLT11*	Myeloid/lymphoid or mixed-lineage leukemia (trithorax homologue, *Drosophila*); translocated to, 11	Translocation partner in leukemia
−0.47	GC35737	AF007140	*ILF3*	Interleukin enhancer binding factor 3, 90 kDa	Posttranscriptional gene expression regulator
−0.462	GC29921	X92106	*BLMH*	Bleomycin hydrolase	Hydrolase involved in pulmonary fibrosis
−0.455	GC36916	AJ223349	*HIRIP3*	HIRA interacting protein 3	Histone-binding protein
−0.448	GC27809	X94232	*MAPRE2*	Microtubule-associated protein, RP/EB family, member 2	Signal transduction, mitotic regulator?
−0.442	GC32318	X65550	*MKI67*	Antigen identified by monoclonal antibody Ki-67	Cell proliferation
−0.442	GC30069	S60415	*CACNB2*	Calcium channel, voltage-dependent, β 2 subunit	Calcium channel
−0.438	GC37769	AF020043	*SMC3*	Structural maintenance of chromosomes 3	Chromosome cohesion during cell cycle.
−0.438	GC34877	D64142	*H1FX*	H1 histone family, member X	Nucleosome condensation
−0.437	GC31579	U62961	*OXCT1*	3-oxoacid CoA transferase 1	Extrahepatic ketone body catabolism
−0.434	GC31159	U37426	*KIF11*	Kinesin family member 11	Prevents centrosome migration and arrest cells in mitosis
−0.434	GC36274	AF106941	*ARRB2*	Arrestin, β 2	Desensitization of G-protein-coupled receptors
−0.433	GC32940	M94630	*HNRNPD*	Heterogeneous nuclear ribonucleoprotein D (AU-rich element RNA binding protein 1, 37kDa)	Binds with high affinity to oncogene and cytokine RNA molecules
−0.432	GC35688	U04810	*TROAP*	Trophinin associated protein (tastin)	Cell adhesion
−0.429	GC33612	L25876	*CDKN3*	Cyclin-dependent kinase inhibitor 3	CDK1and CDK2 inhibitor
−0.428	GC35573	U20979	*CHAF1A*	Chromatin assembly factor 1, subunit A (p150)	Chromatin assembly in DNA replication and DNA repair.
−0.428	GC27963	W84438	*MTERFD2*	MTERF domain containing 2	
0.442	GC31059	AI192108	*RHEB*	Ras homologue enriched in brain	Signal transduction
0.44	GC30387	U59305	*CDC42BPA*	CDC42 binding protein kinase α (DMPK-like) RNA	Signal transduction
0.434	GC28965	AI984786	*BCAP29*	B-cell receptor-associated protein 29	Membrane protein transport from endoplasmic reticulum to Golgi
0.425	GC30348	AI935420	*ARHGAP12*	Rho GTPase activating protein 12	GTPase activator for the Rho-type GTPases
0.423	GC34805	AF027516	*TGOLN2*	Trans-Golgi network protein 2	Membrane traffic to and from trans-Golgi network
0.423	GC37323	D87120	*FAM3C*	Family with sequence similarity 3, member C	Epithelial to mesenchymal transition
0.421	GC27420	X16832	*CTSH*	Cathepsin H	Lysosomal cysteine proteinase
0.411	GC30285	M98343	*CTTN*	Cortactin	Organization of actin cytoskeleton and cell shape
0.409	GC37560	Z29074	*KRT9*	Keratin 9	Cytoskeletal element
0.405	GC39430	AL050220	*KLK13*	Kallikrein-related peptidase 13	steroid-regulated breast cancer marker
0.405	GC31424	AI953179	*TSPAN5*	Tetraspanin 5	Member of the transmembrane 4 superfamily
0.4	GC36737	AI741756	*ATP6V1H*	ATPase, H^+^ transporting, lysosomal 50/57kDa, V1 subunit H	Couples ATPase activity to proton flow.
0.4	GC38134	M62982	*ALOX12*	Arachidonate 12-lipoxygenase RNA	Inflammation, carcinogenesis, membrane remodeling
0.399	GC32483	M32313	*SRD5A1*	Steroid-5-α-reductase, α polypeptide 1 (3-oxo-5 α-steroid delta 4-dehydrogenase α1)	Conversion of testosterone to dihydrotestosterone
0.398	GC29054	AJ133115	*TSC22D4*	TSC22 domain family, member 4	DNA binding transcription factor
0.396	GC30751	D12763	*IL1RL1*	Interleukin 1 receptor-like 1	Interleukin receptor
0.389	GC28792	U32315	*STX3*	Syntaxin 3	Docking of synaptic vesicles
0.389	GC33870	D15049	*PTPRH*	Protein tyrosine phosphatase, receptor type, H	Contact inhibition of cell growth and motility
0.386	GC29420	Z50022	*PTTG1IP*	Pituitary tumor-transforming 1 interacting protein	PTTG1 nuclear translocation
0.384	GC28855	U49837	*CSRP3*	Cysteine and glycine-rich protein 3 (cardiac LIM protein)	Cell differentiation

Positive correlation coefficients indicate direct correlations to log_10_IC_50_ values, negative ones indicate inverse correlations. Information on gene functions was taken from the OMIM database (NCBI, Bethesda, MD, USA) [30] and from the GeneCard database of the Weizman Institute of Science (Rehovot, Israel) [31].

**Table 3:** Separation of clusters of NCI cell lines obtained by hierarchical cluster analysis shown in Figure 5 in comparison to drug sensitivity.

	Partition	Cluster 1	Cluster 2	Cluster 3
sensitive	<−4.160 M	2	19	3
resistant	>−4.160 M	17	6	0

*p* = 2.05 × 10^−5^; The median log_10_IC_50_ value (−5.29 M) for each compound was used as cut-off to separate tumor cell lines as being “sensitive” or “resistant”.

**Table 4:** Molecular docking of scopoletin to NF-κB pathway proteins (details see Figure 8).

Protein	Binding Energy (kcal/mol)	Predicted Inhibition Constant (µM)	Number of Residues Involved in Hydrophobic Interactions	Residues Involved in Hydrogen Bond
I-κB kinase	−7.45 ± 0.00	3.45 ± 0.01	7	Arg144, Lys147
NF-κB-DNA complex	−7.41 ± 0.00	3.69 ± 0.00	4	adenine18, guanine19

## Abbreviations

ABC, ATP-binding cassette; BCRP, breast cancer resistance protein; EGFR, epidermal growth factor receptor; MDR, multidrug resistance; MRP, MDR-related protein; NCI, National Cancer Institute; P-gp, P-glycoprotein; SEAP, secreted embryonic alkaline phosphatase; TLC, thin layer chromatography; TP53, tumor suppressor gene 53; UHPLC, ultra-high-pressure liquid chromatography.

## References

[1] Wahl O., Oswald M., Tretzel L., Herres E., Arend J., Efferth T. (2011). Inhibition of tumor angiogenesis by antibodies, synthetic small molecules and natural products. Curr. Med. Chem..

[2] Efferth T., Grassmann R. (2000). Impact of viral oncogenesis on responses to anti-cancer drugs and irradiation. Crit. Rev. Oncog..

[3] Efferth T., Volm M. (2005). Pharmacogenetics for individualized cancer chemotherapy. Pharmacol. Ther..

[4] Efferth T. (2001). The human atp-binding cassette transporter genes: From the bench to the bedside. Curr. Mol. Med..

[5] Gillet J.P., Efferth T., Remacle J. (2007). Chemotherapy-induced resistance by atp-binding cassette transporter genes. Biochim. Biophys. Acta.

[6] Krishna S., Bustamante L., Haynes R.K., Staines H.M. (2008). Artemisinins: Their growing importance in medicine. Trends Pharmacol. Sci..

[7] Efferth T., Romero M.R., Wolf D.G., Stamminger T., Marin J.J., Marschall M. (2008). The antiviral activities of artemisinin and artesunate. Clin. Infect. Dis..

[8] Efferth T., Zacchino S., Georgiev M.I., Liu L., Wagner H., Panossian A. (2015). Nobel prize for artemisinin brings phytotherapy into the spotlight. Phytomedicine.

[9] Michaelsen F.W., Saeed M.E., Schwarzkopf J., Efferth T. (2015). Activity of artemisia annua and artemisinin derivatives, in prostate carcinoma. Phytomedicine.

[10] Ooko E., Saeed M.E., Kadioglu O., Sarvi S., Colak M., Elmasaoudi K., Janah R., Greten H.J., Efferth T. (2015). Artemisinin derivatives induce iron-dependent cell death (ferroptosis) in tumor cells. Phytomedicine.

[11] Yakasai A.M., Hamza M., Dalhat M.M., Bello M., Gadanya M.A., Yaqub Z.M., Ibrahim D.A., Hassan-Hanga F. (2015). Adherence to artemisinin-based combination therapy for the treatment of uncomplicated malaria: A systematic review and meta-analysis. J. Trop. Med..

[12] Breuer E., Efferth T. (2014). Treatment of iron-loaded veterinary sarcoma by artemisia annua. Nat. Prod. Bioprospect..

[13] Liu X.L., Zhang L., Fu X.L., Chen K., Qian B.C. (2001). Effect of scopoletin on pc3 cell proliferation and apoptosis. Acta Pharmacol. Sin..

[14] Adams M., Efferth T., Bauer R. (2006). Activity-guided isolation of scopoletin and isoscopoletin, the inhibitory active principles towards ccrf-cem leukaemia cells and multi-drug resistant cem/adr5000 cells, from artemisia argyi. Planta Med..

[15] Efferth T., Herrmann F., Tahrani A., Wink M. (2011). Cytotoxic activity of secondary metabolites derived from artemisia annua l. Towards cancer cells in comparison to its designated active constituent artemisinin. Phytomedicine.

[16] Witaicenis A., Seito L.N., da Silveira Chagas A., de Almeida L.D., Luchini A.C., Rodrigues-Orsi P., Cestari S.H., Di Stasi L.C. (2014). Antioxidant and intestinal anti-inflammatory effects of plant-derived coumarin derivatives. Phytomedicine.

[17] Nam H., Kim M.M. (2015). Scopoletin has a potential activity for anti-aging via autophagy in human lung fibroblasts. Phytomedicine.

[18] Efferth T. (2014). Resistance to Targeted abc Transporters in Cancer.

[19] U.S. Department of Agriculture, A.R.S., Dr. Duke’s Phytochemical and Ethnobotanical Databases. http://phytochem.nal.usda.gov/.

[20] Dr. Duke’s Phytochemical and Ethnobotanical Databases. http://dx.doi.org/10.15482/USDA.ADC/1239279.

[21] Breuninger L.M., Paul S., Gaughan K., Miki T., Chan A., Aaronson S.A., Kruh G.D. (1995). Expression of multidrug resistance-associated protein in nih/3t3 cells confers multidrug resistance associated with increased drug efflux and altered intracellular drug distribution. Cancer Res..

[22] Longley D.B., Harkin D.P., Johnston P.G. (2003). 5-fluorouracil: Mechanisms of action and clinical strategies. Nat. Rev. Cancer.

[23] Deeken J.F., Robey R.W., Shukla S., Steadman K., Chakraborty A.R., Poonkuzhali B., Schuetz E.G., Holbeck S., Ambudkar S.V., Bates S.E. (2009). Identification of compounds that correlate with abcg2 transporter function in the national cancer institute anticancer drug screen. Mol. Pharmacol..

[24] Sztiller-Sikorska M., Koprowska K., Majchrzak K., Hartman M., Czyz M. (2014). Natural compounds’ activity against cancer stem-like or fast-cycling melanoma cells. PLoS ONE.

[25] Elfawal M.A., Towler M.J., Reich N.G., Weathers P.J., Rich S.M. (2015). Dried whole-plant artemisia annua slows evolution of malaria drug resistance and overcomes resistance to artemisinin. Proc. Natl. Acad. Sci. USA.

[26] El-Deiry W.S. (1997). Role of oncogenes in resistance and killing by cancer therapeutic agents. Curr. Opin. Oncol..

[27] El-Deiry W.S. (2003). The role of p53 in chemosensitivity and radiosensitivity. Oncogene.

[28] Shetzer Y., Solomon H., Koifman G., Molchadsky A., Horesh S., Rotter V. (2014). The paradigm of mutant p53-expressing cancer stem cells and drug resistance. Carcinogenesis.

[29] Efferth T., Konkimalla V.B., Wang Y.F., Sauerbrey A., Meinhardt S., Zintl F., Mattern J., Volm M. (2008). Prediction of broad spectrum resistance of tumors towards anticancer drugs. Clin. Cancer Res..

[30] National Center for Biotechnology Information. http://www.ncbi.nlm.nih.gov/omim.

[31] Bioinformatics and Biological Computing Unit. http://bioinfo.weizmann.ac.il/cards/index.html.

[32] Kim E.K., Kwon K.B., Shin B.C., Seo E.A., Lee Y.R., Kim J.S., Park J.W., Park B.H., Ryu D.G. (2005). Scopoletin induces apoptosis in human promyeloleukemic cells, accompanied by activations of nuclear factor kappab and caspase-3. Life Sci..

[33] Moon P.D., Lee B.H., Jeong H.J., An H.J., Park S.J., Kim H.R., Ko S.G., Um J.Y., Hong S.H., Kim H.M. (2007). Use of scopoletin to inhibit the production of inflammatory cytokines through inhibition of the ikappab/nf-kappab signal cascade in the human mast cell line hmc-1. Eur. J. Pharmacol..

[34] Yinjun L., Jie J., Yungui W. (2005). Triptolide inhibits transcription factor nf-kappab and induces apoptosis of multiple myeloma cells. Leuk Res..

[35] Wang X., Zhang L., Duan W., Liu B., Gong P., Ding Y., Wu X. (2014). Anti-inflammatory effects of triptolide by inhibiting the nf-kappab signalling pathway in lps-induced acute lung injury in a murine model. Mol. Med. Rep..

[36] Kadioglu O., Efferth T. (2015). Pharmacogenomic characterization of cytotoxic compounds from salvia officinalis in cancer cells. J. Nat. Prod..

[37] Tu Y.Y., Ni M.Y., Zhong Y.R., Li L.N., Cui S.L., Zhang M.Q., Wang X.Z., Liang X.T. (1981). Studies on the constituents of artemisia annua l. (author’s transl). Yao Xue Xue Bao.

[38] Huang L., Liu J.F., Liu L.X., Li D.F., Zhang Y., Nui H.Z., Song H.Y., Zhang C.Y. (1993). Antipyretic and anti-inflammatory effects of artemisia annua l. Zhongguo Zhong Yao Za Zhi.

[39] Luo S.Q., Yuan L., Wu Y.K., Huang J.G. (2013). Effect of fertilization on phenolic components and antioxidant activities of artemisia annua. Zhongguo Zhong Yao Za Zhi.

[40] Yang Y.F., Xu W., Song W., Ye M., Yang X.W. (2015). Transport of twelve coumarins from angelicae pubescentis radix across a mdck-phamdr cell monolayer-an *in vitro* model for blood-brain barrier permeability. Molecules.

[41] Bonelli P., Tuccillo F.M., Borrelli A., Schiattarella A., Buonaguro F.M. (2014). Cdk/ccn and cdki alterations for cancer prognosis and therapeutic predictivity. Biomed. Res. Int..

[42] Shcherba M., Liang Y., Fernandes D., Perez-Soler R., Cheng H. (2014). Cell cycle inhibitors for the treatment of nsclc. Expert Opin. Pharmacother..

[43] Baselga J. (2002). Why the epidermal growth factor receptor? The rationale for cancer therapy. Oncologist.

[44] Lui V.W., Grandis J.R. (2002). Egfr-mediated cell cycle regulation. Anticancer Res..

[45] Lim S.L., Goh Y.M., Noordin M.M., Rahman H.S., Othman H.H., Abu Bakar N.A., Mohamed S. (2016). Morinda citrifolia edible leaf extract enhanced immune response against lung cancer. Food Funct..

[46] Stewart D.J. (2014). Wnt signaling pathway in non-small cell lung cancer. J. Natl. Cancer Inst..

[47] Schwartz S.A., Hernandez A., Mark Evers B. (1999). The role of nf-kappab/ikappab proteins in cancer: Implications for novel treatment strategies. Surg. Oncol..

[48] Efferth T., Koch E. (2011). Complex interactions between phytochemicals. The multi-target therapeutic concept of phytotherapy. Curr. Drug Targets.

[49] Krystof V., Uldrijan S. (2010). Cyclin-dependent kinase inhibitors as anticancer drugs. Curr. Drug Targets.

[50] Lee B., Sandhu S., McArthur G. (2015). Cell cycle control as a promising target in melanoma. Curr. Opin. Oncol..

[51] Klempner S.J., Myers A.P., Cantley L.C. (2013). What a tangled web we weave: Emerging resistance mechanisms to inhibition of the phosphoinositide 3-kinase pathway. Cancer Discov..

[52] Niederst M.J., Engelman J.A. (2013). Bypass mechanisms of resistance to receptor tyrosine kinase inhibition in lung cancer. Sci. Signal..

[53] Namani A., Li Y., Wang X.J., Tang X. (2014). Modulation of nrf2 signaling pathway by nuclear receptors: Implications for cancer. Biochim. Biophys. Acta.

[54] Deeb D., Jiang H., Gao X., Hafner M.S., Wong H., Divine G., Chapman R.A., Dulchavsky S.A., Gautam S.C. (2004). Curcumin sensitizes prostate cancer cells to tumor necrosis factor-related apoptosis-inducing ligand/apo2l by inhibiting nuclear factor-kappab through suppression of ikappabalpha phosphorylation. Mol. Cancer Ther..

[55] Bednarski B.K., Ding X., Coombe K., Baldwin A.S., Kim H.J. (2008). Active roles for inhibitory kappab kinases alpha and beta in nuclear factor-kappab-mediated chemoresistance to doxorubicin. Mol. Cancer Ther..

[56] Chen W., Wang X., Bai L., Liang X., Zhuang J., Lin Y. (2008). Blockage of nf-kappab by ikkbeta- or rela-sirna rather than the nf-kappab super-suppressor ikappabalpha mutant potentiates adriamycin-induced cytotoxicity in lung cancer cells. J. Cell. Biochem..

[57] Choi B.H., Kim C.G., Lim Y., Shin S.Y., Lee Y.H. (2008). Curcumin down-regulates the multidrug-resistance mdr1b gene by inhibiting the pi3k/akt/nf kappa b pathway. Cancer Lett..

[58] Lounnas N., Frelin C., Gonthier N., Colosetti P., Sirvent A., Cassuto J.P., Berthier F., Sirvent N., Rousselot P., Dreano M. (2009). Nf-kappab inhibition triggers death of imatinib-sensitive and imatinib-resistant chronic myeloid leukemia cells including t315i bcr-abl mutants. Int. J. Cancer.

[59] Tracey L., Streck C.J., Du Z., Williams R.F., Pfeffer L.M., Nathwani A.C., Davidoff A.M. (2010). Nf-kappab activation mediates resistance to ifn beta in mll-rearranged acute lymphoblastic leukemia. Leukemia.

[60] Roelandts R. (1984). Mutagenicity and carcinogenicity of methoxsalen plus uv-a. Arch. Dermatol..

[61] Roelandts R. (1987). Carcinogenic risks of phototherapy and photochemotherapy. Hautarzt.

[62] Lake B.G. (1999). Coumarin metabolism, toxicity and carcinogenicity: Relevance for human risk assessment. Food Chem. Toxicol..

[63] Romert L., Jansson T., Curvall M., Jenssen D. (1994). Screening for agents inhibiting the mutagenicity of extracts and constituents of tobacco products. Mutat. Res..

[64] Wagner H., Bauer R., Melchart D., Xiao P.-G., Staudinger A. (2011). Chromatographic Fingerprint Analysis of Herbal Medicines. Thin-Layer and High Performance Liquid Chromatography of Chinese Drugs.

[65] Alley M.C., Scudiero D.A., Monks A., Hursey M.L., Czerwinski M.J., Fine D.L., Abbott B.J., Mayo J.G., Shoemaker R.H., Boyd M.R. (1988). Feasibility of drug screening with panels of human tumor cell lines using a microculture tetrazolium assay. Cancer Res..

[66] Rubinstein L.V., Shoemaker R.H., Paull K.D., Simon R.M., Tosini S., Skehan P., Scudiero D.A., Monks A., Boyd M.R. (1990). Comparison of *in vitro* anticancer-drug-screening data generated with a tetrazolium assay *versus* a protein assay against a diverse panel of human tumor cell lines. J. Natl. Cancer Inst..

[67] Staunton J.E., Slonim D.K., Coller H.A., Tamayo P., Angelo M.J., Park J., Scherf U., Lee J.K., Reinhold W.O., Weinstein J.N. (2001). Chemosensitivity prediction by transcriptional profiling. Proc. Natl. Acad. Sci. USA.

[68] Efferth T., Fabry U., Osieka R. (1997). Apoptosis and resistance to daunorubicin in human leukemic cells. Leukemia.

[69] Scherf U., Ross D.T., Waltham M., Smith L.H., Lee J.K., Tanabe L., Kohn K.W., Reinhold W.C., Myers T.G., Andrews D.T. (2000). A gene expression database for the molecular pharmacology of cancer. Nat. Genet..

[70] Paull K.D., Shoemaker R.H., Hodes L., Monks A., Scudiero D.A., Rubinstein L., Plowman J., Boyd M.R. (1989). Display and analysis of patterns of differential activity of drugs against human tumor cell lines: Development of mean graph and compare algorithm. J. Natl. Cancer Inst..

[71] Wosikowski K., Schuurhuis D., Johnson K., Paull K.D., Myers T.G., Weinstein J.N., Bates S.E. (1997). Identification of epidermal growth factor receptor and c-erbb2 pathway inhibitors by correlation with gene expression patterns. J. Natl. Cancer Inst..

[72] Liu T., Ortiz J.A., Taing L., Meyer C.A., Lee B., Zhang Y., Shin H., Wong S.S., Ma J., Lei Y. (2011). Cistrome: An integrative platform for transcriptional regulation studies. Genome Biol..

[73] Berman H.M., Westbrook J., Feng Z., Gilliland G., Bhat T.N., Weissig H., Shindyalov I.N., Bourne P.E. (2000). The Protein Data Bank. Nucleic Acids Res..

[74] The Protein Data Bank. http://www.rcsb.org/pdb.

